# Sága, a Deep Learning Spectral Analysis Tool for Fungal Detection in Grains—A Case Study to Detect Fusarium in Winter Wheat

**DOI:** 10.3390/toxins16080354

**Published:** 2024-08-13

**Authors:** Xinxin Wang, Gerrit Polder, Marlous Focker, Cheng Liu

**Affiliations:** 1 Wageningen Food Safety Research, Akkermaalsbos 2, 6721 WB Wageningen, The Netherlands; xinxin2.wang@wur.nl (X.W.); marlous.focker@wur.nl (M.F.); 2 Wageningen Plant Research, Wageningen University & Research, 6708 PB Wageningen, The Netherlands; gerrit.polder@wur.nl

**Keywords:** segmentation, wheat ear, hyperspectral, RGB, mycotoxin, deep learning

## Abstract

Fusarium head blight (FHB) is a plant disease caused by various species of the *Fusarium* fungus. One of the major concerns associated with *Fusarium* spp. is their ability to produce mycotoxins. Mycotoxin contamination in small grain cereals is a risk to human and animal health and leads to major economic losses. A reliable site-specific precise *Fusarium* spp. infection early warning model is, therefore, needed to ensure food and feed safety by the early detection of contamination hotspots, enabling effective and efficient fungicide applications, and providing FHB prevention management advice. Such precision farming techniques contribute to environmentally friendly production and sustainable agriculture. This study developed a predictive model, Sága, for on-site FHB detection in wheat using imaging spectroscopy and deep learning. Data were collected from an experimental field in 2021 including (1) an experimental field inoculated with *Fusarium* spp. (52.5 m × 3 m) and (2) a control field (52.5 m × 3 m) not inoculated with *Fusarium* spp. and sprayed with fungicides. Imaging spectroscopy data (hyperspectral images) were collected from both the experimental and control fields with the ground truth of *Fusarium*-infected ear and healthy ear, respectively. Deep learning approaches (pretrained YOLOv5 and DeepMAC on Global Wheat Head Detection (GWHD) dataset) were used to segment wheat ears and XGBoost was used to analyze the hyperspectral information related to the wheat ears and make predictions of *Fusarium*-infected wheat ear and healthy wheat ear. The results showed that deep learning methods can automatically detect and segment the ears of wheat by applying pretrained models. The predictive model can accurately detect infected areas in a wheat field, achieving mean accuracy and F1 scores exceeding 89%. The proposed model, Sága, could facilitate the early detection of *Fusarium* spp. to increase the fungicide use efficiency and limit mycotoxin contamination.

## 1. Introduction

Wheat is the third largest staple food in the world. Diseases, among other factors such as drought, insects, and weeds, are a major factor that could decrease wheat yield and quality [1]. *Fusarium* spp. infection in cereals causes one of the most important diseases in wheat: Fusarium head blight (FHB), and can also result in mycotoxin contamination [2]. Mycotoxins are toxic secondary metabolites produced by fungi. *Fusarium* spp. produce, amongst others, the mycotoxins deoxynivalenol, zearalenone, and fumonisin [3]. Mycotoxin contamination in small grain cereals can lead to food safety incidents, human and animal health problems [4], and economic losses [5,6]. A reliable site-specific precise FHB detection model can facilitate the early warning of *Fusarium* infection and help to limit FHB and mycotoxin formation. Early predictions before flowering could provide management suggestions for farmers such as the spraying of fungicides. Early predictions after flowering and before harvest could give an indication of expected *Fusarium* infection at harvest. Ideally, on-site monitoring and detection allow farmers and advisors to be informed on time and to take locally appropriate measures against *Fusarium* spp. and limit mycotoxin contamination in wheat (although fungal presence does not always mean high mycotoxin concentration, the absence of fungus certainly means the absence of mycotoxins). In addition, the European Union intends to reduce the use of chemical–synthetic plant protection products (csPPPs) by up to 50% by 2030, and an on-site FHB early warning model could facilitate effective pesticide use and benefit sustainable crop protection [7].

Imaging spectroscopy, which combines imaging and spectroscopy, generates spatial information stored in a 2D array of pixels, with spectral information for each pixel stored in the third dimension. This technology allows for the detailed analysis and early detection of Fusarium infections in wheat ears. A previous feasibility study showed *Fusarium* spp. can be detected early using hyperspectral imaging (HSI). Clear spectral differences between the *Fusarium*-infected ear and the healthy ear were observed [8,9,10,11]. An infection can lead to small changes in the structure and/or metabolism of wheat which can be detected several days before the symptoms become visible to the human eye [12,13]. However, these studies used the laboratory testing of wheat ear samples, which are destructive, invasive, and require the transport of samples. In contrast, on-site monitoring for Fusarium head blight (FHB) provides non-invasive, real-time detection and extensive coverage, enabling timely decision making and proactive disease management in wheat fields [13]. In addition, deep learning has proven to be a powerful tool for disease detection based on RGB images or multispectral images [14,15,16,17]. Deep learning involves training neural networks on large datasets to automatically recognize patterns and anomalies. When combined with spectroscopy, deep learning algorithms can analyze complex spectral data to identify subtle differences between healthy and diseased plants. This synergy enhances the accuracy and speed of anomaly detection, making it a valuable approach for the early and precise identification of plant diseases. However, their approaches did not filter out the soil, leaves, and other background information. This information could create heavy noise when using imaging spectroscopy for FHB detection since FHB only occurs on wheat ears. It is essential to segment spectral information only from wheat ears when comparing FHB-contaminated wheat ears and healthy wheat ears. Drawing from previous studies, we anticipate that leveraging imaging spectroscopy coupled with a segmentation approach from deep learning will yield a highly accurate model with consistent results for detecting FHB. To our knowledge, this is the first study to conduct wheat ear segmentation and HSI together for on-site FHB monitoring and detection. This study aimed to develop a site-specific early warning model, Sága, for FHB prediction in winter wheat using imaging spectroscopy and deep learning techniques. Such a model could support decision making by identifying high-risk areas with fungal infection in fields and subsequently provide farmers with early information on where to spray fungicides to limit the use of fungicides and still effectively control FHB.

## 2. Results

### 2.1. Wheat Ear Detection and Segmentation

**Figure 1:**
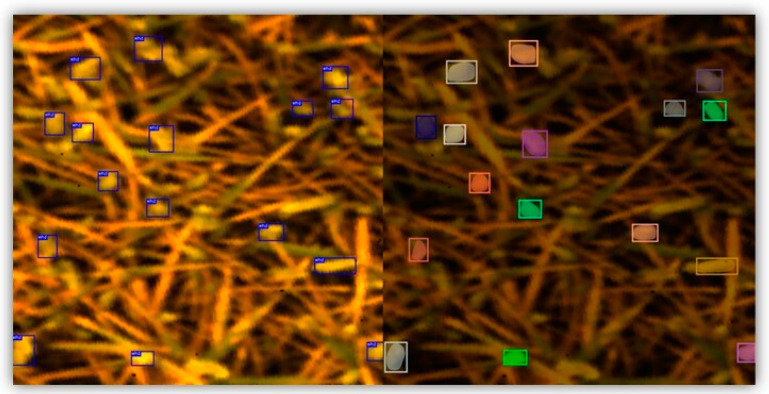
Result of the wheat ear bounding box detection and wheat ear segmentation. The left image shows the detected wheat ears with bounding boxes. The right image shows the segmented wheat ears with colored masks.

Figure 1 shows the outcomes of wheat ear detection and segmentation. Notably, above 90% of the wheat ears (in total, 444 wheat ears) were successfully detected with bounding boxes, as confirmed through visual observation. This achievement showed the effectiveness of the pretrained YOLOv5 model trained on the large GWHD V2 dataset. The results of the wheat ear segmentation are presented in Figure 1 (right). All the detected wheat ears were successfully segmented within the bounding box from the previous step. Although the segmentation process occasionally encompassed areas related to soil within the borders of the mask, the majority of the wheat ear areas were accurately segmented. We considered these segmentation results satisfactory, as they effectively filtered out most irrelevant areas such as wheat leaves and soil. Overall, the successful segmentation of the wheat ears (wheat ear masks) was a significant step in accurately analyzing the spectral properties of the wheat ears.

### 2.2. Spectral Response Difference in the Infected Wheat Ear and Healthy Ear

**Figure 2:**
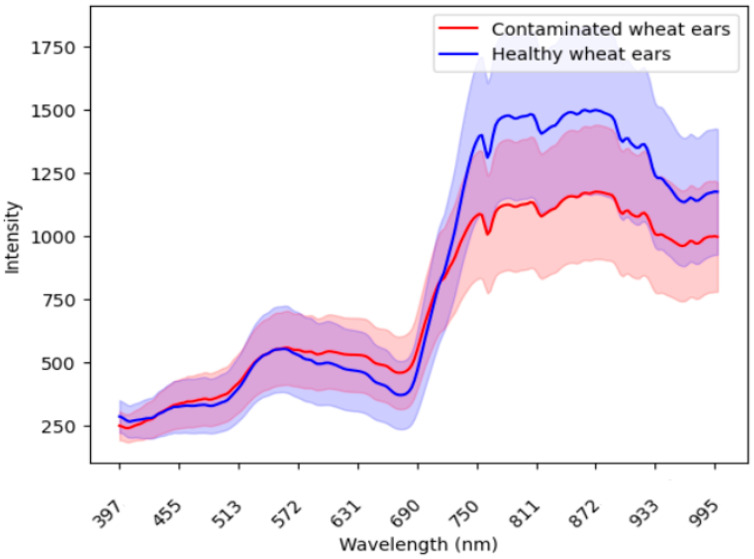
The comparison of the mean wavelength reflectance between the infected ears (red) and healthy ears (blue). The shaded areas around each line represent the standard deviation, which illustrates the variability or dispersion of the reflectance values around the mean.

In the process of using imaging spectroscopy to accurately identify diseases, the difference in spectral signatures between the infected wheat ear and healthy ear was the basis for screening and identifying wheat diseases. Figure 2 compares the mean wavelength reflectance of the wheat ears from the infected spots and healthy spots. Appendix A Figure A3 shows the wavelength reflectance of individual wheat ears. The results demonstrate visually a clear spectral difference between the infected and healthy ears. In the 600–800 nm band, the healthy ears exhibited a distinctive valley and peak in their spectral reflectance, while these features were smoother in the infected ears.

### 2.3. Predictive Model Result

**Figure 3:**
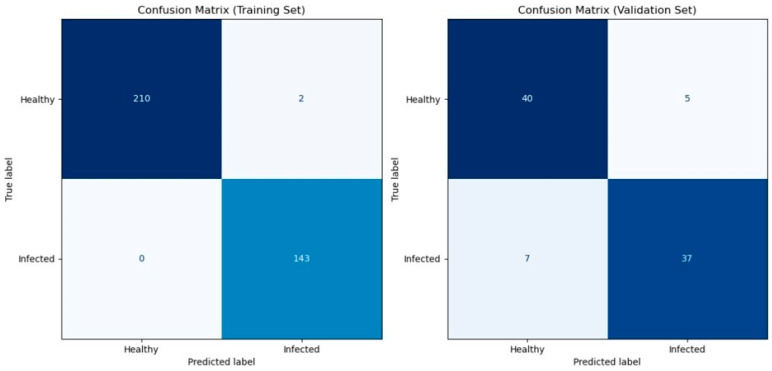
Confusion matrices for model evaluation. Confusion matrices illustrating the performance of the XGBoost classifier on both the training set (**left**) and the validation set (**right**). The matrices display the counts of true positives, true negatives, false positives, and false negatives. The colorbar represents the number of samples in each category. Class labels are “Healthy” (0) and “Infected” (1).

Table 1 shows the cross-validation result of the predictive model based on the spectral information on the wheat ears using the eXtreme Gradient Boosting model. Cross-validation is a robust technique used to evaluate the performance and generalizability of the predictive model by partitioning the data into subsets (folds). Our model achieved high levels of accuracy, precision, recall, and F1 for both the training and validation datasets ranging from 0.82 to 1 for each fold. The mean scores of accuracy, precision, recall, and F1 all exceeded 0.89. This indicates that at least 89% of the infected and healthy wheat ears were correctly classified, and 89% of the infected ears were successfully detected at an early stage. Figure 3 shows the confusion matrix which displays the counts of true positives, true negatives, false positives, and false negatives. Furthermore, the consistency of our model’s performance across the five-fold cross-validation demonstrates its robustness. Finally, we applied the predictive model on one leave-out control plot with 12 wheat ears (unseen data) for external validation, and the model showed 100% prediction accuracy.

**Figure 4:**
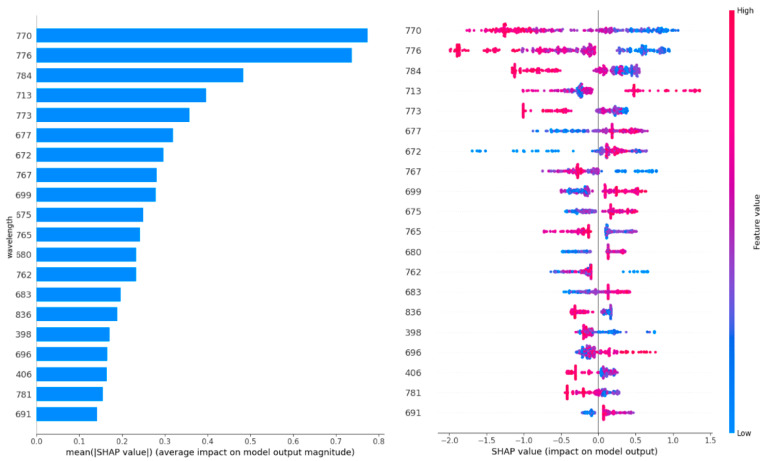
The twenty most important features (wavelengths). The left figure shows the wavelengths sorted based on their importance on the contribution of each feature (wavelength in this case) to the model’s predictive performance. The right figure shows what is the wavelength’s impact on the infected wheat ear.

Figure 4 shows the twenty most important features (wavelength). The wavelengths were sorted based on their importance on the contribution of each feature (wavelength in this case) to the model’s predictive performance. Figure 4 (left) shows a higher SHAP value for a particular wavelength indicating a greater influence of that wavelength on the model’s prediction, suggesting that it is more important for distinguishing between the infected and healthy wheat ears. For example, the wavelengths within the 600–800 nm range contain the most important information (highest SHAP value) to discriminate between classes (e.g., infected vs. healthy wheat ears). Figure 4 (right) shows how the wavelengths reflect the probability of a wheat ear being infected. For example, lower reflectance at a wavelength of 770.11 indicates a higher probability of Fusarium-infected wheat ears. This result is in line with Figure 4.

## 3. Discussion

Using imaging spectroscopy and deep learning techniques for the development of an FHB early warning model is a novel approach in the field of mycotoxin prediction. Imaging spectroscopy can be used to predict the presence of the fungus potentially producing mycotoxins, provide reliable predictions, and reduce the time required for analysis. The development of early warning models is crucial in mitigating the negative impact of FHB and mycotoxin contamination in wheat crops. By identifying areas infected with *Fusarium* ssp., and thus at high risk of mycotoxin contamination, farmers can take necessary measures to prevent or limit contamination, reducing the potential economic losses, safeguarding public health, improving food safety, and benefiting the agricultural industry.

In our study, we applied two deep learning approaches including YOLOv5 and DeepMAC without performing data annotation manually. We did not carry out manual labeling and did not perform deep learning model performance evaluation since (1) the use of the pretrained deep learning models eliminated the need for specific annotation of wheat ears, and the models we utilized had been extensively validated in prior studies, as demonstrated by [18]; (2) the manual labeling of bounding boxes and masks for wheat ears would have entailed considerable labor and effort, and our dataset’s relatively small size rendered it impractical to split it into distinct training, testing, and validation subsets; (3) our primary objective was leveraging a pretrained deep learning model for wheat ear segmentation and extracting spectral information specifically related to the ear region. As such, the evaluation of the accuracy of the segmentation itself was not our primary focus.

We did not apply instance segmentation but applied semantic segmentation to mask wheat ears and further use spectral information for predictive model development. Instance segmentation identifies and separates each individual object in an image, while semantic segmentation classifies each pixel into categories without distinguishing between instances. In this study, semantic segmentation was preferred as it allowed the use of comprehensive spectral data and addressed challenges related to inaccurate GPS locations and the movement of wheat ears, providing a more practical solution. It is difficult to label the specific infected wheat ears and healthy wheat ears separately. This is because we used GPS locations to label the contaminated wheat ears in this study, but GPS locations suffer from certain uncertainties, making it impossible to perfectly match individual wheat ears (wheat ears also move over time). In future studies, we propose to mark the infected ears individually with colored rope, which would be more accurate than relying on GPS locations. That is also the reason that we used buffer zones in our analysis to account for measurement inaccuracies. We also propose that future studies could incorporate real-world testing. By challenging the model in natural environments, we can better understand how it performs under practical conditions that differ from controlled settings. Further studies in this direction would likely reveal insights into the model’s strengths and limitations, providing valuable opportunities for refinement and ultimately leading to more reliable and effective predictive tools for field use.

In addition to using the DeepMAC approach for wheat ear segmentation, other methods can be used to filter out soil. For example, a previous study used an optimized soil-adjusted vegetation index to create a mask that removed soil from the study area but did not remove leaves [17]. Our study chose the DeepMAC approach because we aimed to filter out not only soil but also leaves.

The quality of image acquisition is crucial for accurately identifying wheat ear diseases using deep learning techniques. In our study, the applied TraitSeeker collected images under variable environmental conditions (e.g., wind) that are typically encountered in the field. The sensor took photos of the wheat ears throughout the entire growing area in a short time, providing real-time images that can accelerate wheat disease control and management. Previous studies took close-up photos of individual wheat spikes for wheat ear disease identification [14,15]. As compared to their study, our motorized phenocart/vehicle easily covered the entire wheat field instead of only several parts of it, enabling more efficient data collection.

Our results demonstrated a clear spectral difference between the infected and healthy ears. This finding was consistent with previous studies [9,10,19,20], which have reported similar spectral patterns [20]. The difference in the responses of wheat ears with different severities in the 550–750 nm band could be related to the difference in the pigment content and moisture content in the mesophyll tissue [9]. The severity of wheat diseases was not considered in our study, but previous studies showed that in more severe cases of FHB, the studies indicate a noticeable shift in the distinct spectral differences towards the longer wavelengths within the spectrum [21]. Although the spectral differences are not visible to the naked eye, they provide important information for the early forecasting of *Fusarium* infection.

Previous studies showed that using HSI was more effective than using RGB in FHB early detection. Dammer, Möller [22] found that bands other than red, green, and blue contain some useful information for FHB presence, and the symptoms are difficult to observe in the RGB images at the early stages. Several studies found that the wavelengths near 650 nm were sensitive to wheat FHB [8,19,21,23]. Our findings identify wavelengths of 770, 775, and 783 nm as the most important, which fall into the near-infrared range, indicating that these wavelengths might be particularly effective for detecting FHB beyond visible spectrum indicators. Using imaging spectroscopy, FHB could be effectively detected at 11–14 days after inoculation based on their time series experiments [12,19].

In addition to HSI, multispectral sensors have also been used for wheat canopy information collection, though they provide less data complexity and information [17,24]. Multispectral imaging cameras, which are less expensive and lightweight, are often used for airborne applications on unmanned airborne vehicles (UAVs). Further research can explore using UAVs embedded with multispectral cameras for *Fusarium* detection in wheat with lower equipment costs. However, since multispectral images have fewer bands than those used in our system, the accuracy of detecting FHB may decrease. Therefore, it is recommended to conduct a cost-effectiveness analysis of these two approaches.

The proposed model is a first step towards an early warning model for *Fusarium* spp. In further research, a similar model needs to be developed applicable to dates at least before flowering. At that point, the farmer can take more measures to limit further contamination of the field. One of these measures is the local application of fungicides. Advice on efficient fungicide application can ultimately reduce chemical pollution in the water and soil for a sustainable farming system and reduce crop loss and increase food safety.

## 4. Conclusions

The study developed an early warning model for FHB prediction using innovative *Fusarium* detection techniques, in particular imaging spectroscopy, as inputs, and making use of machine learning and deep learning. First, the results showed that deep learning can automatically detect and segment the ears of wheat. Second, significant differences in spectral reflectance within the 600–800 nm band were observed between the infected and healthy wheat ears. Imaging spectroscopy combined with deep learning can automatically identify if a spot in the wheat field is infected or not with an accuracy of >89%. 

## 5. Method

### 5.1. Field Experiments 

**Figure 5:**
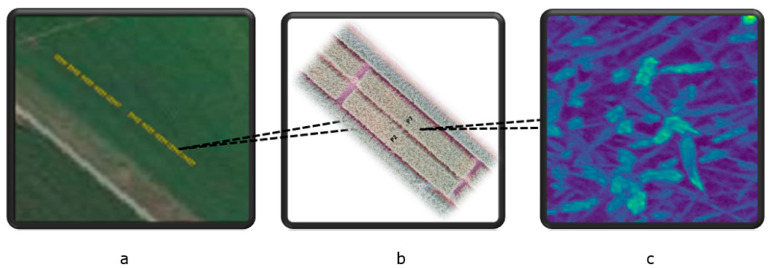
The study area and spectral image acquired by the Netherlands Plant Eco-phenotyping Centre (NPEC) facility TraitSeeker. (**a**): this panel shows the study area, highlighted in yellow, with the upper 10 plots serving as the control plots and the lower 10 plots as the experimental plots. (**b**): this panel provides a zoomed-in view of the two plots for better spatial context. (**c**): This panel displays an example of imaging spectroscopy from one spot within the wheat field, collected from an experimental plot. The image depicts the reflectance at 460 nm, extracted from the full spectral image, with a spatial resolution of 0.2 cm × 0.2 cm.

**Figure A1:**
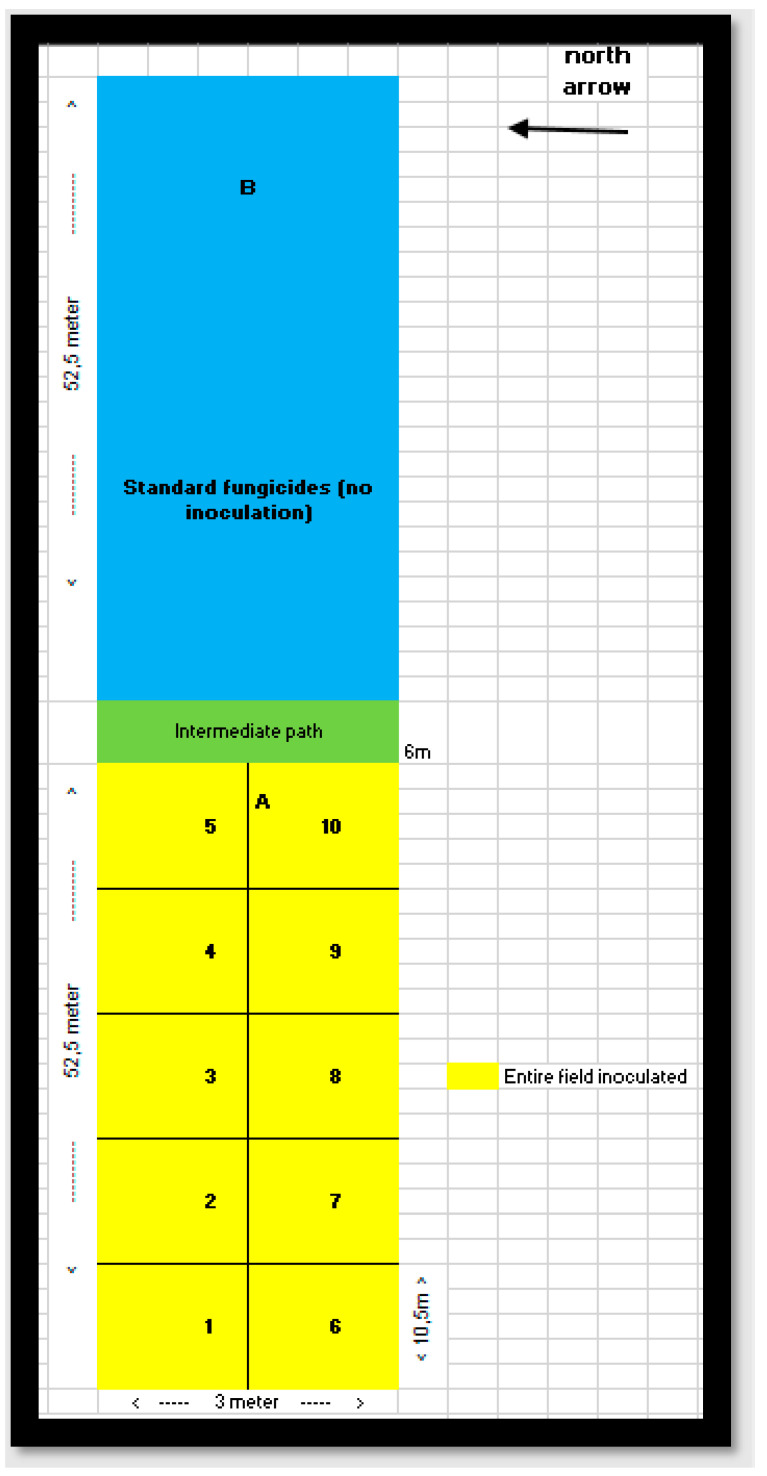
Field experiment plan 2021. Experimental field (yellow): The experimental field, measuring 52.5 m by 3 m, where inoculation was performed to facilitate fungal infection. This field is represented in yellow. Space between fields (green): a 6 m buffer zone separates the inoculated field from the control field to minimize cross-contamination. Control field (blue): The control field, also measuring 52.5 m by 3 m, where fungicide spray was applied and no inoculation was performed. This field is represented in blue.

**Figure A2:**
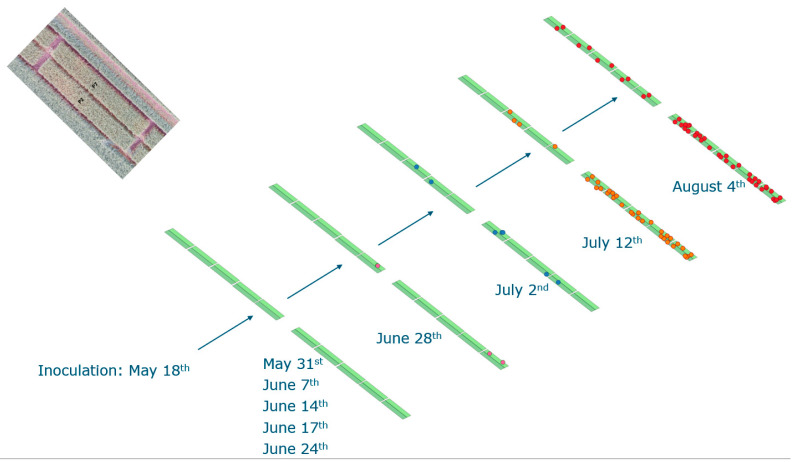
Field experiment plan 2021. Green color shows the wheat fields at different period. Red dots show the observed fusarium head blight spots.

**Figure A3:**
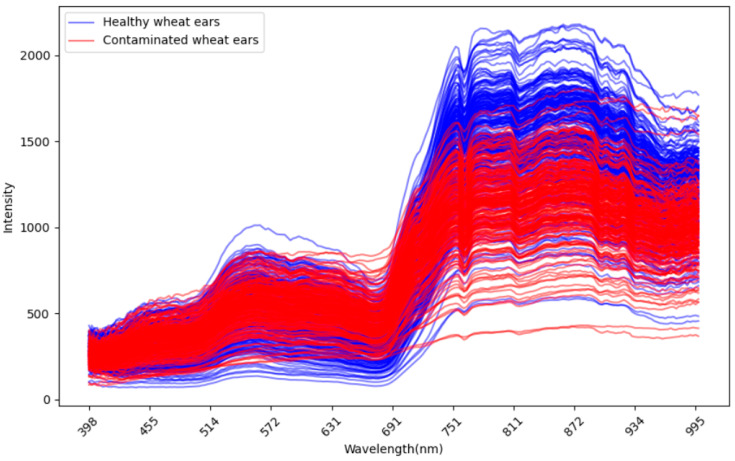
The wavelength reflectance of all the wheat ears between the healthy ear and infected ear.

Winter wheat was grown at the experimental farm Stichting Proefboerderijen Noordelijke Akkerbouw (SPNA) in the north of the Netherlands during the growing season of 2021. The field experiment plan at the SPNA site in 2021 included (1) one experimental field inoculated with *Fusarium* spp. (52.5 m × 3 m) and (2) one control field (52.5 m × 3 m) not inoculated with *Fusarium* spp. and sprayed with standard fungicides (commonly used fungicides that are widely accepted and recommended for use in agriculture to control fungal diseases). We omitted the consideration of transferred fungicides from the control area to the experimental area because we assumed that the amount of fungicides transferred was very small compared to the direct application of fungicides. Both the experimental and control fields were divided into 10 plots. A space of 6 m separated the inoculated and the control fields (Figure A1 and Figure A2 in Appendix A). Figure 5 shows the study area and spectral image acquired by the Netherlands Plant Eco-phenotyping Centre (NPEC) facility TraitSeeker (https://www.wur.nl/en/product/the-netherlands-plant-eco-phenotyping-centre-npec.htm, accessed on 20 January 2024). 

### 5.2. Image Data Acquisition and Data Pre-Processing

**Figure A4:**
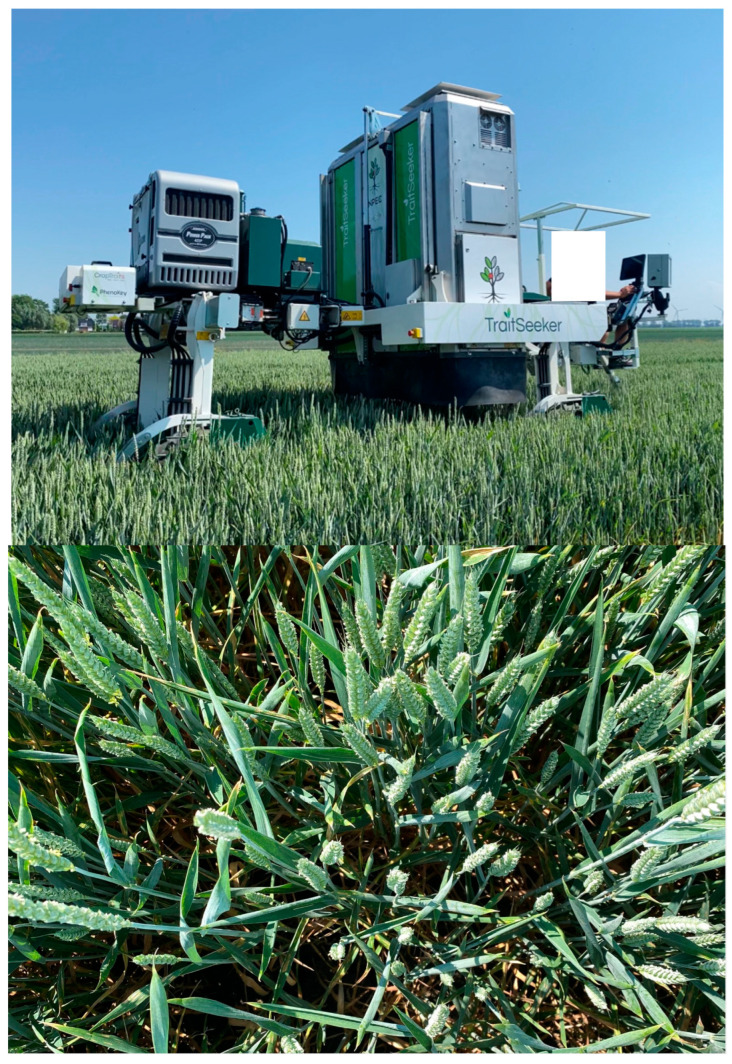
TraitSeeker in the wheat field.

Imaging spectroscopy is often referred to as hyperspectral imaging (HSI), but for the reasons outlined in [25], the term imaging spectroscopy will be used here. Spectral images were taken between May and August 2021, before flowering until harvest. The Netherlands Plant Eco-phenotyping Centre (NPEC) provided a versatile modular facility to carry out accurate high-throughput plant phenotyping with detailed spectral information [25]. The NPEC TraitSeeker device (Figure A4) was used for the imaging of the wheat fields; the TraitSeeker is equipped with a spectral camera covering the Vis-NIR range (400–100 nm) (Specim FX10, SPECIM, Oulu, Finland) and the NIR range (900–1700 nm) (Specim FX17, SPECIM, Oulu, Finland) of the electromagnetic spectrum. The images were acquired in a controlled lighting environment, from a closed box utilizing Tungsten halogen lamps, in order to exclude ambient light. Furthermore, the TraitSeeker is equipped with a high-resolution RGB camera and a Lidar camera (Sick Ranger, St Minneapolis, MN, USA). The images are fused so that for each voxel acquired in 3D space a full reflection spectrum is provided. Spectral cameras used in imaging spectroscopy, unlike conventional RGB cameras, capture data across numerous narrow spectral bands, offering an enhanced spectral resolution. This approach enables a precise identification and analysis of specific wavelengths of light associated with materials or chemical components. Our study focused on using spectral information between 400 nm and 1000 nm, since previous studies observed the spectral difference mainly within this range [12,22].

During the field experiment, image data, along with GPS coordinates, were captured by the TraitSeeker system on 12 July 2021, encompassing both the experimental and control fields. Later, the GPS locations of the identified contaminated spots were recorded through visual inspection on 2 August 2021. Visual inspection was conducted by domain experts based on symptoms such as the bleaching of wheat heads and fungal growth (pink to reddish-brown mold). Subsequently, the GPS coordinates corresponding to the spots labeled as Fusarium head blight (FHB)-contaminated during the visual inspection were utilized as the ground truth for the image data collected on 12 July 2021. This experimental design allows for the early detection of FHB before the entire field is contaminated at harvest. Specifically, during a later visual inspection on 2 August, twice as much contaminated spots were reported than on 12 July, the date used in this study. 

The image data collected on 12 July 2021 were used for developing the predictive model for the early detection of fusarium-contaminated wheat. In the experimental plots, 30 scattered spots were labeled as infected based on visual inspections (the presence of *Fusarium* spp.). In 10 control plots, 30 random scattered spots were labeled as healthy spots (absence of *Fusarium* spp.). All these spots were characterized by their respective GPS locations. For each of the 60 labeled spots, an image with a 20 cm × 20 cm buffer zone (GPS location as central) was created. Later, all the spectral images created were resized to a square format (1024 × 1024 pixels) for further modeling. 

### 5.3. Model Development 

**Figure 6:**
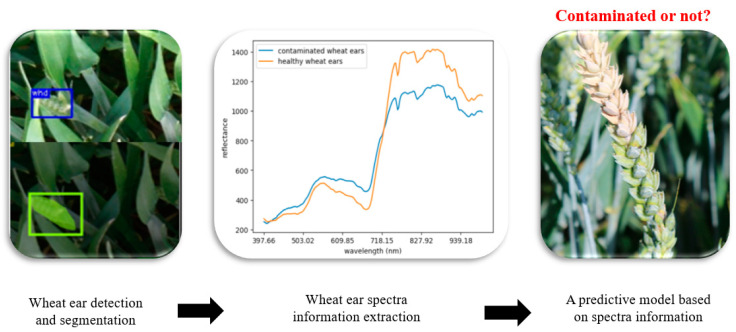
Model development procedure including two modules. The first module consists of the detection and segmentation of wheat ears as well as the extraction of the spectral information from the segmented wheat ears; the second module consists of the creation of a predictive model using the spectral data extracted from the segmented wheat ears.

The model development process comprised two key components: the first module consisted of the detection and segmentation of wheat ears, and the second module consisted of the creation of a predictive model using the spectral data extracted from the segmented wheat ears (as illustrated in Figure 6).

#### 5.3.1. Wheat Ear Detection and Segmentation

Because *Fusarium* spp. mainly infects wheat ears instead of leaves, ear segmentation, by using masks, was performed to filter out other information in the image such as the soil and leaves. Then, the ear masks were applied to spectral images to compare the spectral information between the fusarium-infected spots (with only wheat ears) and the healthy spots (with only wheat ears). 

The deep learning model YOLOv5 was selected for the detection of wheat ears with bounding boxes (https://github.com/ultralytics/yolov5, accessed on 20 January 2024). YOLOv5 is an object detection model that has demonstrated excellent performance in computer vision tasks. It was specifically chosen for its ability to accurately identify and localize wheat ears within images in Global Wheat Challenge 2021 [26,27] (https://www.aicrowd.com/challenges/global-wheat-challenge-2021, accessed on 20 January 2024). We utilized a pretrained YOLOv5 wheat ear detection network in deployment mode for our data analysis. The confidence threshold setting for the bounding boxes was 0.5 and the overlap threshold setting was 0. This pretrained network was trained on a comprehensive image dataset of wheat ears known as the Global Wheat Head Detection (GWHD V2) dataset (http://www.globalwheat.com/, accessed on 20 January 2024). The GWHD_2021 dataset comprises 275,187 wheat heads from 16 institutions distributed across 12 countries, and poor-quality images were eliminated, and the images were labeled with consistency. This dataset provides images with pre-annotated bounding boxes around wheat ears, facilitating the pretraining and deployment of the YOLOv5 model for wheat ear detection. It was crucial for the successful deployment of the deep learning (DL) model to have access to high-quality data, as this directly impacts model performance. Previous research has demonstrated that improving data quality, diversity, and quantity often yields more significant improvements in model efficiency compared to increasing network complexity and depth (Oala et al., 2022). In our study, we opted to exclusively employ the pretrained YOLOv5 model for prediction tasks, abstaining from engaging in the labor-intensive labeling process or directly comparing the performance of different models. This strategic decision was driven by our aim to streamline the deployment process and capitalize on the expertise and robustness inherently embedded in the pretrained model. In addition, we relied on the visual inspection of the model outcomes, trusting in its capability to achieve sufficient detection performance. By applying the pretrained YOLOv5 model to our dataset to detect wheat ears, the yielded bounding boxes laid the foundation for further segmenting the wheat ears. 

For wheat ear segmentation, we applied the state-of-the-art DeepMAC (Deep Mask-heads Above CenterNet) neural network model (Birodkar et al., 2021). Segmentation involves outlining the precise boundaries of objects within an image, providing detailed shape information, whereas detection identifies and locates objects with bounding boxes. YOLOv5 was used for detecting wheat ears with bounding boxes, while DeepMAC was used for segmenting the precise shape of each wheat ear based on the bounding boxes. The DeepMAC model was specifically designed for accurate and efficient segmentation tasks. One significant advantage of utilizing DeepMAC is that it eliminates the need for the manual construction of training masks. The model can learn directly from the bounding boxes generated by the object detection algorithm (YOLOv5 in our case), alleviating the laborious and time-consuming process of manually annotating training data. By leveraging the power of DeepMAC, we were able to obtain precise segmentation masks for each wheat ear in the dataset. These masks provide a pixel-level delineation of the wheat ears, enabling the subsequent analysis and extraction of spectral information Dandrifosse, Ennadifi [18]. Again, in our study, we opted for DeepMAC to abstain from engaging in the labor-intensive labeling process or directly comparing the performance of different models. We relied on the visual inspection of the model outcomes, trusting in its capability to achieve sufficient segmentation performance for further spectral analysis.

The images from the GWHD V2 were RGB images with a format of (1024 × 1024 pixels). Our acquired images were full spectral images, so we created reconstructed RGB images by applying the International Commission on Illumination (CIE) color table to the spectral response. The CIE color table provides a mapping between the spectral values and the corresponding RGB values that can be displayed on a standard RGB monitor [28]. Then, we used the reconstructed RGB images (with the same spatial resolution as the spectral images) as input to the pretrained YOLOv5 model and DeepMAC model to obtain the segmented wheat ears. We qualitatively evaluated these segmentation results by visualization, and we considered it satisfactory if the segmentation results effectively filtered out most irrelevant areas such as wheat leaves and soil. 

#### 5.3.2. Predictive Model Based on Spectral Information of Wheat Ear 

The spectral information of the ears was collected from the spectral images by focusing on the wheat ears. This was achieved by extracting the spectral data from the corresponding pixels within the masked region (the segmentation result from DeepMAC). The spectral values (400 nm–1000 nm) represent the electromagnetic radiation intensities at different wavelengths. Such wavelength from the spots labeled as *Fusarium* ssp.-infected or healthy wheat ears were then compared to show their difference. All these wavelengths serve as the model input for our subsequent modeling and analysis. The eXtreme Gradient Boosting (XGBoost) model [29] was selected to predict the healthy and infected spots. XGBoost is a widely used ensemble learning algorithm which combines the predictions of multiple base learners to generate one overall prediction. Complex relationships between the input features and outputs and the importance of the input features can be learned. Five-fold cross-validations [30] were applied in model training and testing to describe the average model performance and ensure that the model’s performance is robust and reliable. External validation was performed in “unseen” data (meaning data not used for model training and testing) of a 20 cm × 20 cm plot in the experimental field. Feature importance was performed using SHAP (https://shap.readthedocs.io/en/latest/, accessed on 20 January 2024) to analyze the importance of different wavelength for differentiating the infected wheat ear and healthy wheat ear. 

## Figures and Tables

**Table 1:** Cross-validation result of the predictive model. Each row represents a different fold (data in subsets) in the cross-validation process. Accuracy: The ratio of correctly predicted instances (both true positives and true negatives) to the total instances. It measures the overall correctness of the model. Precision: The ratio of true positive predictions to the total positive predictions. It indicates the accuracy of the positive predictions made by the model. Recall: The ratio of true positive predictions to the actual positive instances. It measures the model’s ability to identify all relevant positive cases. F1 score: The harmonic mean of precision and recall. It provides a balanced measure of the model’s performance; especially useful when dealing with imbalanced datasets.

5-Folds	Training Accuracy Scores	Mean Training Accuracy	Training Precision Scores	Mean Training Precision	Training Recall Scores	Mean Training Recall	Training F1 Scores	Mean Training F1 Score	Validation Accuracy Scores	Mean Validation Accuracy	Validation Precision Scores	Mean ValidationPrecision	Validation Recall Scores	Mean Validation Recall	Validation F1 Scores	Mean Validation F1 Score
1-Fold	0.99	0.99	0.98	0.99	1	0.99	0.99	0.99	0.93	0.91	0.93	0.9	0.9	0.88	0.91	0.89
2-Fold	0.99	0.99	1	0.99	0.98	0.99	0.99	0.99	0.93	0.91	0.96	0.9	0.86	0.88	0.91	0.89
3-Fold	1	0.99	0.99	0.99	1	0.99	1	0.99	0.9	0.91	0.82	0.9	0.97	0.88	0.89	0.89
4-Fold	0.99	0.99	0.98	0.99	1	0.99	0.99	0.99	0.89	0.91	0.83	0.9	0.89	0.88	0.86	0.89
5-Fold	0.99	0.99	1	0.99	0.98	0.99	0.99	0.99	0.9	0.91	0.96	0.9	0.79	0.88	0.86	0.89

## Data Availability

The data used in this study are available from the corresponding author upon reasonable request.

## References

[1] Bekele B., Dawit W. (2018). Review on the status and management strategies of Fusarium head blight (Fusarium graminearum) of wheat. Acad. Res. J. Agric. Sci. Res..

[2] Mielniczuk E., Skwaryło-Bednarz B. (2020). Fusarium head blight, mycotoxins and strategies for their reduction. Agronomy.

[3] Ferrigo D., Raiola A., Causin R. (2016). Fusarium toxins in cereals: Occurrence, legislation, factors promoting the appearance and their management. Molecules.

[4] da Rocha M.E.B., Freire F.d.C.O., Maia F.E.F., Guedes M.I.F., Rondina D. (2014). Mycotoxins and their effects on human and animal health. Food Control..

[5] Xia R., Schaafsma A., Wu F., Hooker D. (2020). Impact of the improvements in Fusarium head blight and agronomic management on economics of winter wheat. World Mycotoxin J..

[6] Wilson W., Dahl B., Nganje W. (2018). Economic costs of Fusarium Head Blight, scab and deoxynivalenol. World Mycotoxin J..

[7] Marchand P.A., Robin D. (2019). Evolution of Directive (EC) No 128/2009 of the European Parliament and of the Council establishing a framework for Community action to achieve the sustainable use of pesticides. J. Regul. Sci..

[8] Bauriegel E., Giebel A., Geyer M., Schmidt U., Herppich W. (2011). Early detection of Fusarium infection in wheat using hyper-spectral imaging. Comput. Electron. Agric..

[9] Weber V., Araus J.L., Cairns J.E., Sanchez C., Melchinger A.E., Orsini E. (2012). Prediction of grain yield using reflectance spectra of canopy and leaves in maize plants grown under different water regimes. Field Crops Res..

[10] Liu L., Dong Y., Huang W., Du X., Ma H. (2020). Monitoring wheat fusarium head blight using unmanned aerial vehicle hyperspectral imagery. Remote Sens..

[11] Zhang N., Pan Y., Feng H., Zhao X., Yang X., Ding C., Yang G. (2019). Development of Fusarium head blight classification index using hyperspectral microscopy images of winter wheat spikelets. Biosyst. Eng..

[12] Rieker M.E., Lutz M.A., El-Hasan A., Thomas S., Voegele R.T. (2023). Hyperspectral Imaging and Selected Biological Control Agents for the Management of Fusarium Head Blight in Spring Wheat. Plants.

[13] Thomas S., Kuska M.T., Bohnenkamp D., Brugger A., Alisaac E., Wahabzada M., Behmann J., Mahlein A.-K. (2018). Benefits of hyperspectral imaging for plant disease detection and plant protection: A technical perspective. J. Plant Dis. Prot..

[14] Su W.-H., Zhang J., Yang C., Page R., Szinyei T., Hirsch C.D., Steffenson B.J. (2020). Automatic evaluation of wheat resistance to fusarium head blight using dual mask-RCNN deep learning frameworks in computer vision. Remote Sens..

[15] Bao W., Yang X., Liang D., Hu G., Yang X. (2021). Lightweight convolutional neural network model for field wheat ear disease identification. Comput. Electron. Agric..

[16] Lu J., Hu J., Zhao G., Mei F., Zhang C. (2017). An in-field automatic wheat disease diagnosis system. Comput. Electron. Agric..

[17] Gao C., Ji X., He Q., Gong Z., Sun H., Wen T., Guo W. (2023). Monitoring of Wheat Fusarium Head Blight on Spectral and Textural Analysis of UAV Multispectral Imagery. Agriculture.

[18] Dandrifosse S., Ennadifi E., Carlier A., Gosselin B., Dumont B., Mercatoris B. (2022). Deep learning for wheat ear segmentation and ear density measurement: From heading to maturity. Comput. Electron. Agric..

[19] Bauriegel E., Giebel A., Herppich W.B. (2011). Hyperspectral and chlorophyll fluorescence imaging to analyse the impact of Fusarium culmorum on the photosynthetic integrity of infected wheat ears. Sensors.

[20] Polder G., Van Der Heijden G., Waalwijk C., Young I. (2005). Detection of Fusarium in single wheat kernels using spectral imaging. Seed Sci. Technol..

[21] Zhang D., Wang Q., Lin F., Yin X., Gu C., Qiao H. (2020). Development and evaluation of a new spectral disease index to detect wheat fusarium head blight using hyperspectral imaging. Sensors.

[22] Dammer K.-H., Möller B., Rodemann B., Heppner D. (2011). Detection of head blight (*Fusarium* ssp.) in winter wheat by color and multispectral image analyses. Crop Prot..

[23] Zhang H., Huang L., Huang W., Dong Y., Weng S., Zhao J., Ma H., Liu L. (2022). Detection of wheat Fusarium head blight using UAV-based spectral and image feature fusion. Front. Plant Sci..

[24] Li Y., Cao G., Liu D., Zhang J., Li L., Chen C. (2022). Determination of Wheat Heading Stage Using Convolutional Neural Networks on Multispectral UAV Imaging Data. Comput. Intell. Neurosci..

[25] Polder G., Gowen A. (2021). The hype in spectral imaging. Spectrosc. Eur..

[26] David E., Ogidi F., Smith D., Chapman S., de Solan B., Guo W., Baret F., Stavness I. (2023). Global wheat head detection challenges: Winning models and application for head counting. Plant Phenomics.

[27] David E., Serouart M., Smith D., Madec S., Velumani K., Liu S., Wang X., Pinto F., Shafiee S., Tahir I.S. (2021). Global wheat head detection 2021: An improved dataset for benchmarking wheat head detection methods. Plant Phenomics.

[28] Wyszecki G., Stiles W.S. (2000). Color Science: Concepts and Methods, Quantitative Data and Formulae.

[29] Chen T., He T., Benesty M., Khotilovich V., Tang Y., Cho H., Chen K., Mitchell R., Cano I., Zhou T. (2015). Xgboost: Extreme Gradient Boosting.

[30] Pal K., Patel B.V. Data classification with k-fold cross validation and holdout accuracy estimation methods with 5 different machine learning techniques. Proceedings of the 2020 Fourth International Conference on Computing Methodologies and Communication (ICCMC).

